# Chilean Strawberry Consumption Protects against LPS-Induced Liver Injury by Anti-Inflammatory and Antioxidant Capability in Sprague-Dawley Rats

**DOI:** 10.1155/2015/320136

**Published:** 2015-09-17

**Authors:** Sebastian Molinett, Francisca Nuñez, María Alejandra Moya-León, Jessica Zúñiga-Hernández

**Affiliations:** ^1^Instituto de Ciencias Biológicas, Universidad de Talca, 2 Norte No. 685, Talca, 3465548 Maule, Chile; ^2^Departamento de Farmacología, Escuela de Medicina, Universidad de Talca, 2 Norte No. 685, Talca, 3465548 Maule, Chile

## Abstract

The Chilean strawberry fruit has high content of antioxidants and polyphenols. Previous studies evidenced antioxidant properties by *in vitro* methods. However, the antioxidant effect and its impact as functional food on animal health have not been evaluated. In this study, rats were fed with a Chilean strawberry aqueous extract (4 g/kg of animal per day) and then subjected to LPS-induced liver injury (5 mg/kg). Transaminases and histological studies revealed a reduction in liver injury in rats fed with strawberry aqueous extract compared with the control group. Additionally, white strawberry supplementation significantly reduced the serum levels and gene expression of TNF-*α*, IL-6, and IL-1*β* cytokines compared with nonsupplemented rats. The level of F2-isoprostanes and GSH/GSSG indicated a reduction in liver oxidative stress by the consumption of strawberry aqueous extract. Altogether, the evidence suggests that dietary supplementation of rats with a Chilean white strawberry aqueous extract favours the normalization of oxidative and inflammatory responses after a liver injury induced by LPS.

## 1. Introduction

The native white Chilean strawberry (*Fragaria chiloensis* spp. chiloensis) is a native species from Chile and the maternal progenitor of the commercial strawberry (*Fragaria *x* ananassa* Duch) [1]. The Mapuche people cultivated this plant as a nutritive food, consumed as fresh or dried fruit or prepared for medicinal purposes [2]. During the last decade, in Chile there has been an increasing interest in expanding the culture and production of* F. chiloensis* to gain a niche in the local and global market, as well as take advantage of a resurgent interest in new crops [3].

Berry fruits are known to have an antioxidant effect that could prevent different pathological conditions [4]. Strawberry fruits possess a remarkable nutritional composition due to their high content of micronutrients such as folates, minerals, and vitamins and are also a rich source of phenolic constituents [5]. Particularly, white Chilean strawberries have been described as a good source of phenolic antioxidants [6]. Strawberry extracts have higher antioxidant activity compared with other common consumable fruits [7]. The antioxidant properties of strawberries are mainly due to phenolic compounds [8] and this capability has been proven to be effective against superoxide and hydrogen peroxide radicals [9].* In vitro* studies have shown that white Chilean strawberry fruit has high free radical scavenging activity [1, 8]. Despite the potential of this fruit as antioxidant, there is a lack of information about the* in vivo* effects on the oxidative status in animal models. Nevertheless, previous studies have reported that the consumption of commercial strawberry fruit provides beneficial health effects. The supplementation of diet with strawberry fruit, as a source of antioxidants, reduced the level of blood lipids and the oxidative damage of LDL in hyperlipidemic subjects [10]. Furthermore, a hypocholesterolemic effect and a decrease in lipid peroxidation were reported [11]. In another study, antiplatelet function was evidenced by* in vitro* and* in vivo* approaches [12]. On the other hand, anti-inflammatory effect has been evidenced [13], in which strawberries are very effective inhibiting cyclooxygenase (i.e., COX2), a key enzyme in inflammation pathway and a target for different drugs. In addition, recent studies suggest that strawberry polyphenols might have a role in cancer prevention and treatment [5]. Considering that the available knowledge about beneficial health effect of commercial strawberry fruit and the converse scenario of white Chilean strawberry fruit has a different phenolic profile, it becomes relevant to gain insight into its potential properties as functional food.

The lipopolysaccharide (LPS) is a major glycolipid component of the outer cell wall of Gram-negative bacteria, made up from a polysaccharide O-chain and a biologically active lipid-A moiety embedded within the bacterial membrane [14]. In rats, LPS triggers the rise of cytokines and reactive oxygen species (ROS) such as superoxide, hydroxyl radicals, and peroxynitrite [15]. The endotoxemia induced by LPS is characterized by the injury of various organs including liver, kidney, and brain [14]. Most of the toxicities observed in LPS-induced liver injury (and systemic) have been attributed to inflammatory mediators produced by activated macrophages, including tumour necrosis factor-*α* (TNF-*α*) and interleukins IL-1*β*, IL-6, IL-8, and IL-12 [16]. These toxic mediators can induce lipid peroxidation, oxidative damage, and depletion of intracellular stored antioxidants [17], creating a prooxidative and inflammatory status. Therefore, the present study was designed to evaluate the effects of an aqueous solution of strawberry fruit orally administrated on the inflammatory response and oxidative status in a LPS-injured liver. Biochemical changes and histological parameters were analysed.

## 2. Material and Methods

### 2.1. Animals

Male Sprague-Dawley rats (200–250 g) were obtained from the animal facility of University of Talca. The animals were housed on 12 h light-dark cycles at 25°C. Animals received standard animal rodent chow and water* ad libitum.* Care of animals and the experimental protocols of this study were approved by the Institutional Animal Use Committee of the University of Talca and conducted in accordance with the recommendations of the European Union regarding animal experimentation (Directive of the European Council 86/609/EC).

### 2.2. *F. chiloensis* Fruit Extract

**Figure 1:**
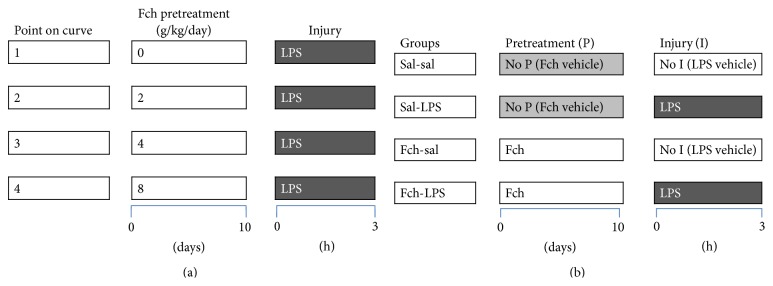
Experimental protocol for Fch pretreatment. (a) Curve doses-response: animals were given 10 days Fch (2, 4, and 8 g/kg/day). (b) Protocol of animal design groups after analysis of curve doses-response. Animals were given 10 days Fch (4 g/kg/day). After the period of Fch administration, group of control (light grey bars) and Fch-treated (dark grey bars) animals were subject to LPS-induced liver injury, thus conforming the experimental groups. Blood and liver samples were obtained at 3 hours of LPS challenger (or LPS-vehicle control).

Ripe fruits of* Fragaria chiloensis* ssp.* chiloensis* (record number: 5537 in plant list http://www.theplantlist.org/) were collected in December 2013 from a commercial orchard at Manzanar Alto, Purén, IX Region, Chile (38°2′30.2′′S, 73°8′38.4′′W). After harvest, the fruit was immediately cold transported to the Institute of Biological Sciences at University of Talca, and after freezing it under liquid nitrogen it was stored at −80°C until use. Ripe* F. chiloensis* fruit was homogenized in distilled water (0.5 g/mL) with the help of an Ultraturrax (5 min at 4000 rpm) on an ice bath. The rats were fed twice a day with* F. chiloensis* extracts at the daily dosages of 2, 4, and 8 g/kg of animal per day (for the doses-response curve). All the administrations were performed with fresh aqueous extracts (Figure 1(a)).

### 2.3. Animal Preparation and LPS-Induction of Liver Injury

Groups of rats were orally supplemented for 10 days with the aqueous* F. chiloensis *extract 2 g/kg of animal twice a day, that is, 4 g/kg/day of strawberry (Figure 1(b)), while control rat groups received isovolumetric amounts of saline solution (0.9% NaCl). At day 10 the animals were challenged to a LPS sepsis induced by a single intraperitoneal rat injection of 5 mg/kg LPS (lipopolysaccharide endotoxin,* Escherichia coli*, serotype 0111:B4, Sigma-Aldrich, San Luis, MO, USA) from* E. coli* 8 [18] dissolved in 0.9% NaCl. These generate four experimental groups: (a) sal-sal (saline pretreatment plus a saline LPS vehicle), (b) sal-LPS (saline pretreatment plus LPS inoculum), (c) Fch-sal (*F. chiloensis* extract during pretreatment plus LPS vehicle), and (d) Fch-LPS (Fch pretreatment plus LPS inoculum). Five to six animals composed each experimental group.

After 3 hours of LPS challenge the animals were anesthetized with an intraperitoneal injection (1 mL/kg) of zolazepam chlorhydrate (25 mg/mL) and tiletamine chlorhydrate (25 mg/mL) (Zoletil 50; Virbac S/A, Carros, France). Blood samples were obtained by cardiac puncture for serum analysis. Liver samples were taken from the medial lobes, frozen in liquid nitrogen, and stored at −80°C for further analysis.

### 2.4. Histological Analysis

Liver samples of each animal were fixed in phosphate-buffered formalin, dehydrated in increasing ethanol concentrations, and embedded in paraffin. Thereafter, sections of tissue were cut at 5 *μ*m on a rotatory microtome (Leica Ultracut, Solms, Germany), mounted on clean glass slides, and dried overnight at 37°C. Sections were cleared, hydrated, and stained with haematoxylin and eosin for histomorphological assessments. Blind histopathological evaluation was performed in at least ten randomly chosen microscopic fields of all histological slides (three to five slides per each animal assayed). All tissue sections were examined in a Nikon Eclipse 50i microscope (Tokyo, Japan) for characterization of histopathological changes. Photographs were digitalized using micrometrics SE premium 2.8 version camera and software for microscopy.

### 2.5. Determination of Transaminases, Cytokines, F2-Isoprostanes, and Glutathione Assays

Transaminases levels, AST (aspartate transaminase) and ALT (alanine transaminase), were measured using a specific diagnostic kit (SpinReact, Girona, Spain) and expressed as international units/L. ELISA kits were used for assessment of serum levels (pg/mL) of TNF-*α*, IL-6, IL-1*β*, and IL-10 cytokines (Biosource International, Camarillo, CA, USA) and F2-isoprostanes (Cayman Chemical, Ann Arbor, MI, USA). GSH and GSSG contents (*μ*mol/g liver) were measured using a specific glutathione assay kit (Cayman Chemical).

### 2.6. RNA Extraction and Quantitative Real-Time PCR

Total RNA was isolated from liver samples by the guanidinium thiocyanate-phenol-chloroform extraction method (Trizol, Life Technologies, Carlsbad, CA, USA). The extracted RNA was treated with DNAse (Turbo DNA-free kit, Ambion, Life Technologies) to eliminate DNA contamination. The RNA quantity and purity were estimated at 260/280 nm by NanoDrop 1000 Spectrophotometer (Thermo Scientific, Waltham, MA, USA) and the cDNA was synthesized with a first strand cDNA synthesis kit (Thermo Scientific, Waltham, MA, USA). Quantitative PCR was performed in a Stratagene Mx3000P (Agilent Technologies, Santa Clara, CA, USA). Each reaction consisted of 20 *μ*L containing 2 *μ*L of (1 : 10 diluted) cDNA, 1 *μ*L of primer mix 10 *μ*M, 10 *μ*L of Sybr Green PCR Master Mix 2X (Stratagene, Agilent Technologies), and nuclease free water to reach the final reaction volume. The cycling conditions were 1 cycle of denaturation at 95°C for 5 min, followed by 40 two-segment cycles of amplification (95°C/15 sec, 60°C/45 sec) and a final melting cycle (95°C/1 min, 55°C/30 sec, and 95°C/30 sec). From each experimental group (5-6 animals) three liver samples were randomly chosen, from which RNA was independently isolated and cDNA prepared; qPCR runs were performed using three technical replicates and the mean was used for further analysis.

The primers used for quantitative PCR analysis were TNF-*α* forward GGTTCCGTCCCTCTCATACA, reverse AGACACCGCCTGGAGTTCT; IL-6 forward ACTGGTCTGTTGTGGGTGGT, reverse AGTTGCCTTCTTGGGACTGA; IL-1*β* forward GCTGTGGCAGCTACCTATGCTACCTATGTCTTG, reverse AGGTCGTCATCATCCCACGAG; IL-10 forward GCTGTGGCAGCTACCTATGTCTTG, reverse AGGTCGTCATCATCCCACGAG; GAPDH forward GGCCTCTCTCTTGCTCTCAGTA, reverse TTCTCAAGCTCATTTCCTGGTA. The expression levels were normalized by ΔΔCT method using GAPDH as reference gene and the relative expression was calibrated and assigned value one to sal-sal condition.

### 2.7. Statistical Analysis

All values correspond to means ± standard error of the mean (SEM) or standard deviation (SD). The data were evaluated with GraphPad Prism 6 software (La Jolla, CA, USA). The statistical significance of differences for each parameter among the groups was evaluated by Tukey's test for unpaired data or one-way ANOVA. A *p* value of less than 0.05 was considered significant.

## 3. Results

### 3.1. Protective Effect of* F. chiloensis* in Acute LPS-Induced Liver Injury

**Figure 2:**
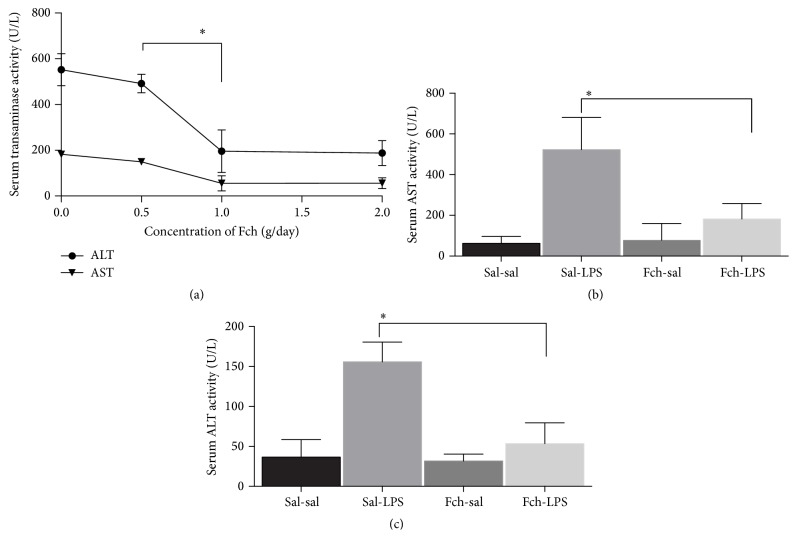
Effect of* F. chiloensis* supplementation on serum transaminase levels after LPS challenge. (a) Changes in response to different* F. chiloensis* fruit dosage (Fch). Values correspond to mean ± SEM of at least 4–6 animals per experimental group. Rats were fed daily with the indicated amount of* F. chiloensis* fruit extract (Fch) for 10 days and then subjected to LPS challenge, which consisted in one intraperitoneal injection of LPS (5 mg/kg). ((b) and (c)) Changes in serum AST and ALT in response to LPS challenge. Each bar corresponds to mean ± SEM of 5 to 6 different animals per experimental group. Significant differences between the groups are indicated by asterisk (*p* < 0.05, one-way ANOVA, and Tukey's multiple comparison test).

It has been reported that a LPS challenge induces a liver injury [19, 20]. A significant increase in serum AST and ALT transaminases was observed as expected in our experimental conditions (Figure 2). The effect of several Fch feeding dosages (0.5, 1, and 2 g per day of Fch extract) was tested for their capability to decrease AST and ALT exacerbation after 3 h of LPS challenge (Figure 2(a)). A strong reduction in transaminases was found at a dosage of 4 g/kg/day (2 doses of 2 g/kg of animal Fch) or higher dosages. A dosage of 4 g/kg/day of fruit extract was employed in further experiments.

**Figure 3:**
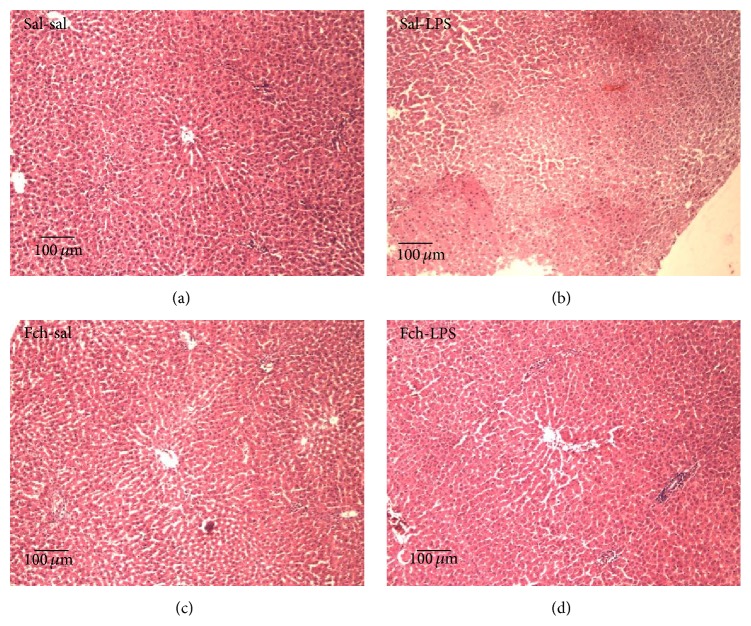
Effects of* F. chiloensis* supplementation in liver morphology after LPS challenge. Representative liver sections stained with haematoxylin-eosin from different experimental conditions: sal-sal (saline pretreatment plus a saline LPS vehicle), sal-LPS (saline pretreatment plus LPS inoculum), Fch (Fch pretreatment plus LPS vehicle), and Fch-LPS (Fch pretreatment plus LPS inoculum). A total of 5-6 different animals per experimental group were evaluated; original magnification ×100; scale bar is 100 *μ*m.

The supplementation of* F. chiloensis* extract on diet prior to LPS challenge avoids the exacerbation in serum AST (Figure 2(b)) and ALT (Figure 2(c)) activities, and values comparable to those in nonchallenged rats were determined. Control rats subjected to LPS single injection exhibited a 7.8- and 4.3-fold increase (*p* < 0.05) in serum AST and ALT activities, respectively, in comparison to sal-sal treatment, and importantly this increment in activity was suppressed by Fch supplementation. In agreement with these data, liver histological assessments showed normal liver morphology in sal-sal and Fch-sal groups (Figures 3(a) and 3(c), resp.). Changes in liver architecture (disruption) in extensive areas, hepatocytes necrosis, and inflammatory infiltrates were observed in LPS challenged animals (Figure 3(b)). On the contrary, the livers of Fch-LPS group displayed normal architecture, with minimal or absence of necrosis (Figure 3(d)). This indicates that morphological changes induced by LPS are prevented by Fch supplementation.

### 3.2. *Fragaria chiloensis* Reduces the Inflammatory Damage Induced by LPS

Cytokines pro- and anti-inflammatory were determined by ELISA in serum samples obtained from rats subjected to LPS challenge. In addition, gene expression analyses of the same cytokines were performed in liver samples by RT-qPCR.

**Figure 4:**
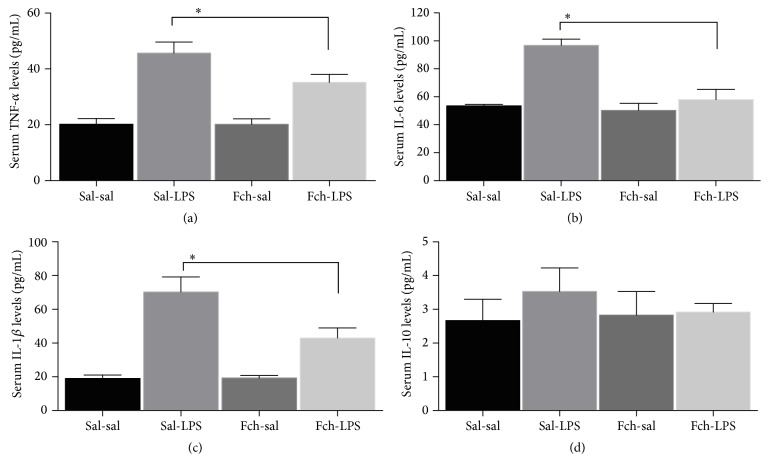
Effects of* F. chiloensis* on serum cytokine levels in response to LPS challenge. TNF-*α* (a), IL-6 (b), IL-1*β* (c), and IL-10 (d) levels. Each bar corresponds to mean ± SEM of 5-6 different animals per experimental group. Significant differences between the groups are indicated by asterisk. *p* < 0.05, one-way ANOVA, and Tukey's multiple comparison test.

The levels of serum TNF-*α* (Figure 4(a)), IL-6 (Figure 4(b)), and IL-1*β* (Figure 4(c)) increased in response to LPS challenge by 2.3-, 1.8-, and 3.7-fold compared to sal-sal treatment (*p* < 0.05). In animals supplemented with* F. chiloensis* fruit extract the increment of cytokines pro- and anti-inflammatory in response to LPS challenge is prevented. There were nonsignificant changes in IL-10 in response to LPS treatment (Figure 4(d)).

**Figure 5:**
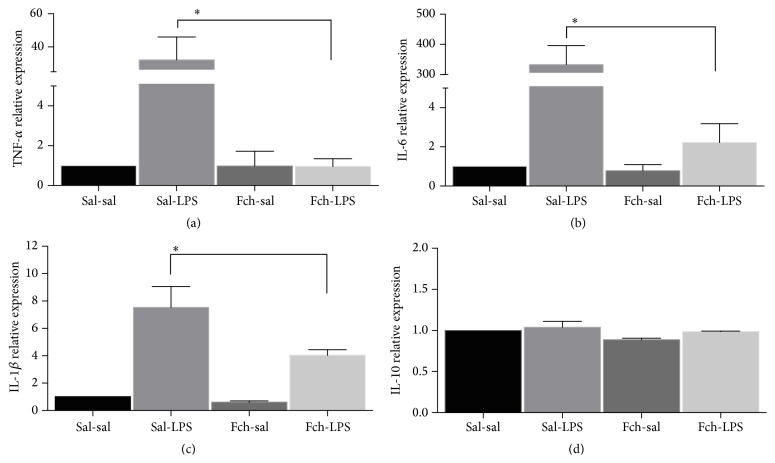
Effect of Fch supplementation on cytokine gene expression in liver tissue. TNF-*α* (a), IL-6 (b), IL-1*β* (c), and IL-10 (d) mRNAs were determined by RT-qPCR and normalized to GAPDH. Each bar corresponds to mean ± SEM of 5-6 different animals per experimental group. Significant differences between the groups are indicated by asterisk. *p* < 0.05, one-way ANOVA, and Tukey's multiple comparison test.

On the other hand, the levels of TNF-*α*, IL-6, and IL-1*β* mRNA were also increased in LPS treated animals and nonsupplemented (48-, 318.9-, and 14.4-fold, resp., *p* < 0.05) compared to nontreated (sal-sal) (Figures 5(a), 5(b), and 5(c)). Furthermore, the increment in TNF-*α*, IL-6, and IL-1*β* transcripts in response to LPS was prevented in animals supplemented with* F. chiloensis* aqueous fruit extract: values were significantly decreased by 33.9-, 149-, and 1.8-fold, respectively, in the Fch-LPS group compared to sal-LPS group. No changes in mRNA levels of IL-10 were observed among the different experimental groups (Figure 5(d)).

### 3.3. Liver Oxidative Stress Related Parameters Induced by LPS Are Diminished by* F. chiloensis* Supplementation

**Figure 6:**
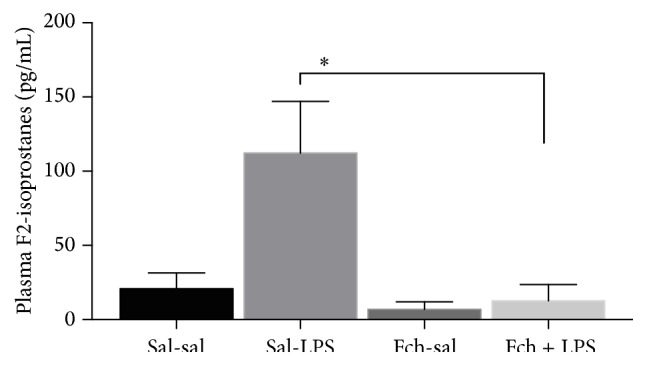
Effect of Fch supplementation on the plasma levels of 8-isoprostanes after hepatic LPS challenge. Values correspond to means ± SEM of 5-6 different animals per experimental group. Significant differences between the groups are indicated by asterisk. *p* < 0.05, ANOVA, and the Tukey multiple comparison test.

The levels of plasma 8-isoprostanes increased in response to LPS challenge (8.7-fold) compared to sal-sal treatment (Figure 6). In animals supplemented with* F. chiloensis* aqueous extract the increment in 8-isoprostanes in response to LPS challenge is avoided.

**Figure 7:**
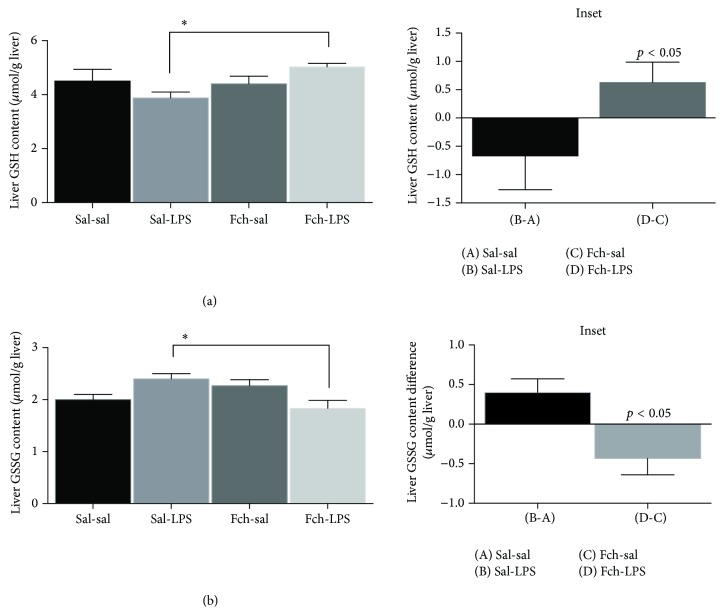
Effects on serum glutathione levels in response to LPS challenge. (a) Reduced (GSH) and (b) oxidized glutathione (GSSG) levels. Values represent the means ± SEM of 5-6 different animals per experimental group. Significant differences between the groups are indicated by asterisk (*p* < 0.05, ANOVA, and Tukey's multiple comparison test). Inset: net effects of the LPS-induced liver injury in the content of glutathione with or without pretreatment with Fch. (B)-(A) correspond to the difference between serum levels in LPS and the control condition and (D)-(C) correspond to the difference between Fch-sal and Fch-LPS group of rats.

The content of hepatic reduced glutathione (GSH) diminished (23%) in response to LPS challenge compared to control (sal-sal) treatment (Figure 7(a)). The GSH content in animals supplemented with* F. chiloensis* aqueous extract was similar to controls without LPS inoculum; however a significant enhancement in GSH is observed after LPS challenge (Figure 7(a), inset).

On the other hand, liver glutathione disulphide (GSSG) levels increased (23.6%) in response to LPS challenge compared to sal-sal treatment (Figure 7(b)). However, in response to LPS the animals pretreated with* F. chiloensis* achieved a net reduction in GSSG levels compared to nonsupplemented rats subjected to LPS challenge (Figure 7(b), inset).

## 4. Discussion

The focus of this study was to investigate the acute effects of LPS-induced hepatic damage on the inflammatory response and oxidative stress status and the possible protection offered by the oral administration of an aqueous extract of the native Chilean white strawberry. Typically, a portion of the released endotoxin (LPS) is absorbed into the portal circulation and delivered to the liver, where it is quickly cleared by intrahepatic Küpffer cells [21]; thus the liver plays a central role in the regulation of entry and metabolism of LPS inoculum. The model of LPS has been previously shown to result in a reproducible acute inflammatory response, with increments in cytokines and oxidative stress, mitochondrial dysfunction, and early biochemical changes associated with organ dysfunction, similar to the changes observed in human [22]. Mostly, the hepatic and systemic sepsis (toxicities) induced by LPS have been attributed to an overproduction of reactive oxygen species (ROS) and the release of chemical mediators such as peroxide, nitric oxide, and proinflammatory cytokines (massive release of TNF-*α*, IL-1*β*, and IL-6), which are all formed as a result of the binding of LPS to Toll-like receptor 4 (TLR-4) on the surface of Küpffer cells [16, 23]. As a response, hepatocytes strive to clear LPS, which is followed by inflammation, hepatocellular apoptosis, and even necrosis [24].

Oxidative stress is a well-known mechanism of LPS-induced hepatic injury and produces a redox imbalance which may result in depletion of endogenous antioxidant such as the antioxidant enzymes, alteration of GSH redox status [14], and generation of 8-isoprostanes, free radical-catalysed products from lipid peroxidation (arachidonic acid derivatives) [25]. The antioxidant defence system and its equilibrium become necessary, especially during infection or against an oxidative insult. There are reports in which plant extracts, infusions, or isolated compounds have been used to reverse or prevent the hepatotoxicity and the oxidative stress generated by LPS, with beneficial effects associated with antioxidant and anti-inflammatory properties:* Rooibos* aqueous [14],* Eucalyptus globulus* leaf extract [26], polyphenol extract of* Hibiscus sabdariffa* [27, 28], and fermented Barley extract [29]. In this study, we reported that a single injection of LPS resulted in hepatic injury as indicated by the elevation in the levels of serum ALT and AST, described as circulating markers of hepatocyte injury. The hepatic marker enzymes are cytoplasmic in physiological condition but are usually leaked into circulation when liver damage occurs due to an alteration in membrane integrity [14, 30]. Results from the current study showed that supplementation of diet with the aqueous extract of native Chilean white strawberry for 10 days prior to the LPS challenge at a dose of 4 g/kg/day diminished the induced damage in the liver. Several studies have suggested that the phytochemical content and antioxidant/free radical scavenging effect of fruits and vegetables contribute to their protective effect against chronic and degenerative diseases [31]. The contribution of vitamin C to the total antioxidant activity of twelve different fruits analysed was estimated as being <15% [7]. Strawberry extracts were found to have higher antioxidant activity than extracts from other studied fruits. However, vitamin C is not the only one that contributes to the antioxidant activity of fruits and vegetables. The antioxidant properties of strawberries have been shown to be mainly due to high content of phenolic compounds more than to vitamin C [31–33]. Wang and Jiao [9] evidenced that strawberry aqueous extract exhibited a high level of antioxidant capacity against free radical species including superoxide radicals, hydrogen peroxide, hydroxyl radicals, and singlet oxygen; also it is important to mention that actually there does not exist any study that links the hepatoprotection with the antioxidant capability of strawberry.

The polyphenolic composition, specifically flavonoid and phenolic compounds, of different strawberries cultivars differs amongst them [32, 34, 35]. In addition, the content of phenolic compounds differs significantly amongst strawberry species and subspecies and determines the free radical scavenging activity of these fruits [8]. The main phenolic compounds in the Chilean white strawberry are ellagic acid and hexahydroxydiphenolic acid-based hydrolysable tannins and procyanidins, as well as flavonol glycosides from quercetin and kaempferol [6] (Table 1). The protective effect of* F. chiloensis* extract observed in our study may be due to the ability of its phenolic content to stabilize and maintain the integrity of the hepatocyte as well as repair damage tissue by stimulating hepatocyte regeneration and modulation of Küpffer cell activation.

Küpffer cells have well-defined roles in the coordination of hepatic inflammation in a number of diseases [36, 37]. During endotoxemia, LPS is detected by Toll-like receptor 4 (TLR4) on sentinel cell (endothelium, Küpffer cells, etc.) that initiates the subsequent inflammatory response, including neutrophil recruitment to the liver [37]. LPS binding to Küpffer cells initiates a cascade of events that upregulates expression of the inflammatory cytokines including TNF-*α* stimulating the production of ROS and reactive nitrogen intermediates by activated macrophages causing liver damage due to the oxidative stress. Additionally, LPS induces migration of activated polymorphonuclear leukocytes (PMNs) extending and keeping the liver injury [37]. In the present study, LPS challenge significantly increased levels of TNF-*α*, IL-1*β*, and IL-6, and the oral administration of* F. chiloensis* prior to LPS challenge was able to reduce the increment in serum cytokines and the expression level in the liver of related genes at a comparable level compared to that in nonchallenged animals. Interestingly, these results are consistent with the data reported for green tea polyphenols [38], quercetin [39], and resveratrol [40]. These studies have shown an anti-inflammatory effect associated with the control of signal transduction, with the reduction in the expression of proinflammatory proteins (i.e., COX-2), inducible tumour necrosis factor-*α* (TNF-*α*), and nitric oxide synthase.

On the other hand, we did not find any difference in the levels of IL-10 cytokines, although IL-10 is produced by macrophages as a negative-feedback mechanism to dampen uncontrolled production of inflammatory cytokines and excessive inflammation during infection. IL-10 is a potent anti-inflammatory cytokine with a broad effect on both innate and adaptive immune system [41]. IL-10 could downregulate TNF-*α* as well as other cytokines by suppressing their gene expression in an autocrine-like feedback loop [42]. As our observations showed that the levels of IL-10 were similar between control and treated groups, these could indicate that the inhibition of the other cytokines observed by* F. chiloensis *supplementation may be independent of the activation of the feedback mediated by IL-10. Thus, we speculate that the protective effect detected is due to the antioxidant capability of* F. chiloensis* extract and another molecular mechanism associated with LPS signalling pathway. This idea is reinforced by the observed GSH/GSSG normalization and anti-inflammatory effect.

The impairment of the antioxidants defence system is a critical step in LPS-induced injury. Evidence has shown that LPS insult is characterized by change in tissue and circulating antioxidant enzyme's level and antioxidant molecules, like GSH [14]. We found a modulation of the antioxidant system in the LPS-challenged rats consuming* F. chiloensis*, with the normalization of GSH/GSSG ratio and a depletion of 8-isoprostane levels. Reduced glutathione (GSH) is the major nonprotein thiol in plant and animal cells. GSH is recognized as a highly effective antioxidant compound, as its scavenging and antioxidant properties allow the neutralization of ROS species. It is essential for the regulation of a variety of cellular functions [14], and a consistent and sufficient GSH level can prevent LPS-induced damage [43]. The ability to improve the GSH/GSSG ratio shown by* F. chiloensis* can be ascribed to the capability of the different fruit antioxidant components to quench free radicals, to upregulate the synthesis of GSH, and to restore the antioxidant status, results comparable with the total* in vitro* scavenging activity determined previously (Table 1). The glutathione regulation observed is consistent with the low levels of 8-isoprostanes observed in LPS challenged rats supplemented with* F. chiloensis* extract. Isoprostanes comprise a group of free radical-catalysed peroxidation products of arachidonic acid in a COX (cyclooxygenase) independent mechanism. They are seen as oxidative stress markers and potentially mediate some of the adverse effects of oxidant injury [25, 44]. ROS have been reported to drive inflammatory cytokines responses by a mechanism that comprises nuclear factor kappa B transcription factor (NF*κ*B) that regulates several cytokines including IL-1*β* and IL-6 [45]. Some antioxidants such as MitoQ, MitoE, and melatonin can reduce IL-1*β* and IL-6 levels and decrease NF*κ*B activation, effect observed in a rat model with acute LPS sepsis [22]. A recent study suggested that total flavonoids extracted from flowers of* Abelmoschus manihot* (L.) Medic (TFA) protected mice against carbon tetrachloride- (CCl_4_-) induced liver injury through antioxidant stress and anti-inflammatory effects, which decreased the MDA level and elevated the content of GSH in the liver as compared to those in the CCl_4_ group. Meanwhile, the inflammatory mediators (e.g., TNF-alpha, IL-1*β*, and NO) were inhibited by TFA treatment both at the serum and mRNA levels [46]. On the other side, quercetin alleviates inflammation after short-term treatment in high-fat-fed (HFD) mice. Increased nuclear import of NF*κ*B and elevated expressions of proinflammatory markers were further manifestations in the HFD group. All these changes were reversed in the quercetin-treated groups with significant improvement of antioxidant activity compared to the HFD group [47]. These data are consistent with the fact that NF*κ*B could be the transcription factor that regulates the pathways of hepatoprotection observed in* F. chiloensis* supplementation. The Pi3K/AKT/NF*κ*B signalling pathway is involved in LPS-induced injury; moreover, the NF*κ*B signalling pathway is known to play a critical role in sepsis-associated organ failure [18, 48]. It has been reported that the flavonoid apigenin inhibits LPS-induced inflammation through inhibition of NF*κ*B activation by hypophosphorylation of ser536 in the p65 subunit in* in vivo* model [48]. Thus, further investigation may be warranted to design the relation between NF*κ*B and the liver protection observed in this study.

Alternatively, the potential blocking of lipopolysaccharide binding protein (LBP) mechanism might be related to the anti-inflammatory observed results, where LBP is an acute phase protein which plays an important role in lipopolysaccharide (LPS) signalling and innate immunity and their level in acute liver injury and liver failure may significantly affect the physiological derangements that are observed in acute liver failure, liver transplantation, common bile duct ligation, alcoholic liver injury, and hemorrhagic shock [49, 50]. However, there have been limited studies of the role of LBP in animal and human models of severe acute liver injury [49]. It is generally accepted that low levels of LBP augment the cell's response to LPS, whereas high levels of LBP have been shown to inhibit cell responses to LPS. Cells with LBP knockout became refractory to proinflammatory cytokines and other inflammatory stimuli (LPS and palmitate). This effect was mediated through decreased NF*κ*B signalling and reversed by exogenous LBP [51]. Also the administration of LBPK95A (LBP inhibitory peptide) attenuates liver injury after acetaminophen-induced hepatotoxicity administration as evidenced by reductions in serum transaminase levels and decreased centrilobular necrosis with poor Küpffer cell function; less liver injury was noted in the absence of LBP [50].

## 5. Conclusions

Our study demonstrates, for the first time, the hepatoprotective activity of a native Chilean strawberry aqueous extract, which maintained hepatocellular membrane structural integrity, attenuated hepatic oxidative stress, and inhibited inflammatory response in LPS-induced liver injury. These effects were achieved by normalization of liver parameters, redox status (GSH/GSSG ratio and decrease of isoprostanes), and downregulation of cytokines (TNF-a, IL-1*β*, and IL-6). The results of this study support the dietary supplementation with Chilean strawberry as a novel noninvasive strategy to protect the liver and other organs against LPS injury. Further experiments are being conducted by our research group.

## Figures and Tables

**Table 1:** Total content of anthocyanins, flavonols, ellagic acid (mg/100 g fresh weight), and scavenging activity in strawberry fruits.

	*Fragaria chiloensis* ssp. *chiloensis*	*F*. x *ananassa* cv. Chandler
Total anthocyanins^*∗*^	2,2	27,9
Total flavonols^*∗*^	1,78	1,76
Total ellagic acid^*∗*^	157,29	37,45
% Scavenging activity (DPPH)^*∗∗*^	79,3	64,2

^*∗*^Modified from Simirgiotis et al., 2009 [1].

^*∗∗*^Modified from Simirgiotis and Schmeda-Hirschmann, 2010 [6], and Cheel et al., 2007 [8].
